# Class-level DILI saturation and hERG-driven risk stratification among 2024–2025 FDA-approved kinase inhibitors: an in silico multi-platform safety analysis

**DOI:** 10.1007/s10637-026-01615-1

**Published:** 2026-05-02

**Authors:** Nihan Küçük Yılmaz, Veysel Baskın

**Affiliations:** 1 Department of Medical Pharmacology, Faculty of Medicine, Yalova University, Yalova, Turkey; 2 Department of Medical Pharmacology, Faculty of Medicine, Hitit University, Çorum, Turkey

**Keywords:** Kinase inhibitors, In silico safety profiling, HERG cardiotoxicity, Drug-induced liver injury, Weighted ToxPi, CYP3A4-mediated drug–drug interaction

## Abstract

Newly approved kinase inhibitors (2024–2025) have limited post-approval safety data. We developed a weighted ToxPi framework to prioritize multi-endpoint liabilities spanning hERG cardiotoxicity, DILI, Ames genotoxicity, and CYP3A4-mediated drug-drug interaction potential. Thirteen compounds (12 KIs approved in 2024–2025 plus imatinib as a historical reference anchor) were profiled using ADMETlab 3.0, vNN-ADMET, and SwissADME (formula inputs) and integrated into a weighted composite (wToxPi = 0.30 × hERG + 0.30 × DILI + 0.20 × Ames + 0.20 × CYP3A4). Robustness was assessed across weighting schemes and sensitivity analyses addressing fusion-recoding scenarios, CYP3A4 platform asymmetry, and combination-regimen confounding. DILI signal saturation dominated the cohort: 12 of 13 compounds showed DILIcon ≥ 0.80, limiting hepatic discrimination and shifting differentiation to hERG and Ames variability. Sevabertinib (0.860), Zongertinib (0.840), and Lazertinib (0.775) ranked highest, whereas Mirdametinib (0.080) was the structural outlier. Ranking remained stable: ADMETlab-only and thresholded majority-vote alternatives preserved the principal structure (Spearman *ρ* = 0.780 and 0.812), whereas soft-label pseudo-probability mapping showed near-identity concordance (*ρ* = 0.978); a broader sensitivity grid (*p* = 0.60–0.90) confirmed structural stability (*ρ* = 0.929–0.995). Exploratory FAERS findings provided supportive post-marketing context. This framework provides transparent, reproducible, hypothesis-generating prioritization of newly approved KIs. Class-level DILI saturation reinforces the need for close hepatic surveillance in pharmacovigilance interpretation regardless of composite rank; hERG and Ames provide the main inter-compound discriminatory signal. Sevabertinib, Zongertinib, and Lazertinib ranked highest, whereas Mirdametinib was the structural outlier; for Inavolisib and Tovorafenib, endpoint-specific hepatic surveillance remains warranted irrespective of composite rank. The model should support pharmacovigilance prioritization rather than replace clinical monitoring or individualized risk assessment.

**Supplementary Information:**

The online version contains supplementary material available at 10.1007/s10637-026-01615-1.

## Introduction

Kinase inhibitors (KIs) have transformed oncology through mechanism-based, biomarker-driven treatment. Since imatinib’s approval for CML in 2001, more than 80 small-molecule KIs have received FDA approval [1]. Despite this progress, the conserved architecture of kinase ATP-binding pockets facilitates off-target interactions producing diverse and clinically consequential toxicity profiles. The 2024–2025 approval cycle yielded HER2 TKD inhibitors (Sevabertinib, Zongertinib), EGFR exon 20 insertion inhibitors (Sunvozertinib), ROS1/ALK/PI3K inhibitors, and pediatric agents (Tovorafenib, Mirdametinib)—a cohort characterized by limited post-approval real-world pharmacovigilance data [2–4].

Four mechanistically distinct safety liabilities are of particular concern for this drug class. hERG (Kv11.1) channel inhibition, mandated for assessment by the ICH E14/S7B framework, may precipitate Torsades de Pointes and is a central proarrhythmic concern [5, 6]. DILI is the leading cause of post-market withdrawal and black-box warnings, with KIs disproportionately implicated through lipophilicity-driven reactive metabolite generation [7, 8]. Ames genotoxicity is a critical endpoint for chronically administered agents [9]. CYP3A4-mediated DDIs directly affect the polypharmacy-burdened oncology patient population. Animal models show approximately 55% sensitivity for human DILI [10], reinforcing the clinical utility of multi-platform in silico profiling as a hypothesis-generating complement to clinical monitoring.

Individual in silico platforms employ distinct training sets, molecular descriptors, and algorithms. Consensus modeling—aggregating multiple independent predictions—reduces variance and broadens applicability-domain coverage [11]. The Toxicological Priority Index (ToxPi) translates multi-domain toxicological data into a weighted composite score with explicit endpoint slices, supporting transparent prioritization [12, 13]. The composite indicator construction methodology applied here follows established principles for normalization, weighting, and aggregation of heterogeneous domain scores [13]. Importantly, the framework described here constitutes heuristic prioritization, not a calibrated clinical risk model; it supports pharmacovigilance hypothesis generation rather than clinical decision-making.

This study contributes (1) systematic multi-platform in silico profiling of 2024–2025 FDA-approved KIs across four distinct safety endpoints; (2) a reproducible consensus scoring methodology with explicit fusion heuristics and quantified limitations; (3) sensitivity-validated wToxPi prioritization across three weight schemes with rank-based robustness analysis; and (4) directional contextual plausibility mapping against FDA label safety signals, with explicit acknowledgment of combination-regimen confounding for four agents.

## Materials and methods

### Interpretive scope

The wToxPi framework is a transparent, reproducible heuristic prioritization tool—not a calibrated clinical risk model. It does not incorporate pharmacokinetic parameters (free plasma Cmax, AUC) or patient-specific modifiers. All results should be interpreted as hypothesis-generating signals for pharmacovigilance prioritization, not as clinical risk estimates or prescribing guidance.

### Study design and compound selection

Twelve KIs approved by the FDA between January 2024 and November 2025, plus imatinib (approved in 2001) as a historical multi-endpoint reference anchor, were included. Imatinib was not used to tune, calibrate, or define any formula parameter; its inclusion constitutes a qualitative face-validity check only and does not influence the prioritization of the 2024–2025 cohort. Four agents—Lazertinib, Avutometinib, Defactinib, and Inavolisib—were approved in or are primarily evaluated in combination regimens; clinical safety signal comparisons for these agents carry greater interpretive uncertainty (see “Discussion”).

### In silico platforms

Five publicly accessible platforms were used: ADMETlab 3.0 (continuous probability scores, 0–1) [14]; vNN-ADMET (binary Yes/No, coded 1/0) [15]; Pred-hERG 5.0 (categorical hERG classification; contextual reference only) [16]; ProTox 3.0 (organ toxicity predictions; contextual reference only) [17]; and SwissADME (CYP3A4 inhibition and physicochemical descriptors) [18]; all accessed March 2026. ADMETlab 3.0, vNN-ADMET, and SwissADME contributed to consensus score derivation; Pred-hERG 5.0 and ProTox 3.0 served as contextual reference tools only. All compounds fell within the Pred-hERG 5.0 applicability domain except Mirdametinib (flagged throughout). During the March 2026 access period, no version-level changes were evident from the publicly available model-version, training-description, or applicability-domain information for ADMETlab 3.0 and ProTox 3.0. Platform outputs were therefore treated as version-consistent within the study window.

### Consensus score derivation and fusion heuristic

hERGcons = mean(ADMETlab_hERG, vNN_hERG); DILIcon = mean(ADMETlab_DILI, vNN_DILI); Amescons = mean(ADMETlab_Ames, vNN_Ames); CYP3A4cons = mean(ADMETlab_CYP3A4, vNN_CYP3A4, Swiss_CYP3A4). Full raw outputs, consensus scores, and an endpoint/platform/coding data dictionary are provided in Online Resource 3 (Tables S3.1 and S3.3). Arithmetic-mean fusion of continuous ADMETlab scores with binary vNN/SwissADME outputs is a pragmatic heuristic: binary “Yes” contributes exactly 0.5 to any two-platform consensus regardless of the ADMETlab value, introducing step-function effects and variance compression, which were further examined in sensitivity analyses and rank-based robustness assessment.

### Weighted ToxPi formula, weight justification, and prioritization-tier thresholds

Primary scheme (W1): wToxPi = 0.30 × hERGcons + 0.30 × DILIcon + 0.20 × Amescons + 0.20 × CYP3A4cons. hERG and DILI receive the highest equal weight given the acute proarrhythmic risk (ICH E14) [5] and the leading post-market withdrawal burden; Ames and CYP3A4 receive a secondary equal weight as mechanistically relevant liabilities whose clinical consequences are more often managed through monitoring and dose adjustment. Prioritization tiers (High ≥ 0.75; Moderate 0.50–0.74; Low < 0.50) were defined a priori as internal visualization categories only—not validated clinical risk strata. Three weight schemes were pre-specified: W1 primary, W2 equal (0.25 each), W3 cardiac/hepatic heavy (0.40/0.40/0.10/0.10). A step-by-step worked example of the wToxPi calculation for Sevabertinib, including all platform inputs, consensus score derivation steps, and the final composite score under all three weight schemes, is provided in Online Resource 1.

### Sensitivity analyses

Three supplementary analyses were performed. (i) Binary–continuous fusion sensitivity: the primary arithmetic-mean strategy was compared with an ADMETlab-only continuous scenario, a thresholded majority-vote scenario (ADMETlab binarized at 0.5; positive if ≥ 1 platform for 2-platform endpoints, ≥ 2 for CYP3A4), and a soft-label pseudo-probability scenario (Yes = 0.75, No = 0.25), with a broader grid (*p* = 0.60–0.90) additionally examined. (ii) CYP3A4 platform asymmetry: the 3-platform consensus was replaced by a 2-platform alternative (ADMETlab + vNN only) and rank/tier changes were examined. (iii) Combination-regimen sensitivity: agents approved primarily in combinations were excluded and rankings re-expressed for the retained subset without score recalculation. Full results are in Online Resource 4 (Tables S4.1A, S4.1B, S4.2, S4.3).

### Rank-based robustness analysis

Each compound was ranked 1–13 within each endpoint, and Spearman’s correlation between wToxPi rank ordering and summed endpoint rank ordering was computed as a non-parametric robustness check that reduces sensitivity to binary-coding distortion.

### Exploratory FAERS-based contextual review

Publicly available FAERS records were retrieved for all study compounds (observation windows: FDA approval date to 31 March 2026; imatinib: 10 May 2001 to 31 March 2026). Total cases, DILI-compatible hepatic preferred terms, strict QT-specific preferred terms, and broader cardiac preferred terms were summarized descriptively. FAERS data served as contextual support only and were not incorporated into wToxPi computation (Online Resource 4, Table S4.4).

## Results

### Study compounds and approval characteristics

The study set comprised 12 KIs approved in 2024–2025, together with imatinib as a historical reference anchor, spanning HER2/EGFR, PI3K, ALK/ROS1, CSF1R, RAF, and MEK pathway inhibitors (Table 1). Four agents were approved in or are primarily evaluated in combination regimens: Avutometinib and Defactinib (mandatory combination), Lazertinib (with amivantamab), and Inavolisib (with palbociclib/fulvestrant). Clinical safety data for these agents reflect combination pharmacology and cannot be attributed to the indexed compound without disaggregated single-agent data; accordingly, contextual plausibility mapping in Table 3 is more uncertain for these four compounds.

**Table 1:** Study compounds: target, indication, and FDA approval date

No	Drug (brand)	Target	Indication	FDA approval
1	Sevabertinib (Hyrnuo)	HER2 TKD inhibitor	HER2 TKD mutation + non-squamous NSCLC (post-prior systemic therapy)	19 Nov 2025
2	Zongertinib (Hernexeos)	HER2 TKD inhibitor	HER2 TKD mutation + non-squamous NSCLC	08 Aug 2025
3	Sunvozertinib (Zegfrovy)	EGFR exon20ins inhibitor	EGFR exon 20 insertion + metastatic NSCLC (post-platinum)	02 Jul 2025
4	Taletrectinib (Ibtrozi)	ROS1 TKI	ROS1 + locally advanced/metastatic NSCLC	11 Jun 2025
5	Avutometinib ^a^ (Avmapki)	MAPK pathway inhibitor	KRAS-mutated recurrent LGSOC (combination with defactinib)	08 May 2025
6	Defactinib ^a^ (Fakzynja)	FAK/PYK2 inhibitor	KRAS-mutated recurrent LGSOC (combination with avutometinib)	08 May 2025
7	Vimseltinib (Romvimza)	CSF1R inhibitor	Symptomatic TGCT	14 Feb 2025
8	Mirdametinib (Gomekli)	MEK1/2 inhibitor	NF1-associated symptomatic plexiform neurofibromas	11 Feb 2025
9	Ensartinib (Ensacove)	ALK inhibitor	ALK + locally advanced/metastatic NSCLC (inhibitor-naive)	18 Dec 2024
10	Inavolisib ^a^ (Itovebi)	PI3Kα inhibitor	PIK3CA-mut HR +/HER2 − advanced breast cancer (combination with palbociclib/fulvestrant)	10 Oct 2024
11	Lazertinib ^a^ (Lazcluze)	EGFR TKI (3rd gen)	EGFR exon19del/L858R NSCLC, 1st-line (combination with amivantamab)	19 Aug 2024
12	Tovorafenib (Ojemda)	Type II RAF kinase inhibitor	Relapsed/refractory pediatric LGG (BRAF fusion/V600)	23 Apr 2024
13	Imatinib (Gleevec) anchor	BCR-ABL/PDGFR/KIT	Multiple indications; multi-endpoint historical reference anchor	10 May 2001 (Ref)

^a^Combination-regimen approval: in silico scores represent single-compound profiling only, clinical safety signals reflect combination pharmacology and cannot be attributed to the indexed compound alone. *LGSOC*, low-grade serous ovarian cancer, *NSCLC* non-small cell lung cancer, *TGCT* tenosynovial giant cell tumor; *LGG*, low-grade glioma, *NF1* neurofibromatosis type 1

### Physicochemical properties

Molecular weights ranged from 405.20 to 584.01 Da, with six compounds exceeding the 500 Da Lipinski threshold [19], consistent with the beyond-Rule-of-5 physicochemical space often occupied by contemporary oral targeted agents [20]. Several compounds showed predicted low GI absorption; these platform-derived estimates should be interpreted cautiously when considering exposure-related toxicity. Full physicochemical data are provided in Online Resource 2.

### Endpoint-level patterns and discriminatory power

The dominant finding was near-universal DILI signal saturation: 12 of 13 compounds showed DILIcon ≥ 0.80 (range 0.094–1.000), with Mirdametinib as the sole outlier. This pattern is consistent with class-level KI characteristics—including lipophilicity, extensive CYP3A4 involvement, and reactive metabolite potential—rather than compound-specific hepatotoxic liability [21]. Consequently, DILI contributes substantially to absolute wToxPi scores but provides limited inter-compound discrimination; rank differences among non-outlier compounds principally reflect hERGcons and Amescons variability.

hERGcons showed meaningful spread (0.026–0.994): several EGFR/HER2-targeted agents clustered at high values, whereas Tovorafenib, Mirdametinib, and Inavolisib showed distinctly low values. Amescons showed the widest bidirectional spread (0.011–0.993), with Sevabertinib, Zongertinib, and Imatinib at the upper end and Defactinib and Ensartinib at the lower end. Full endpoint-level data are provided in Online Resource 3, Table S3.1.

### Weighted ToxPi scores and rank stability

Sevabertinib (0.860), Zongertinib (0.840), and Lazertinib (0.775) ranked highest among newly approved agents, whereas Mirdametinib (0.080) was the structural outlier, consistent with its non-ATP-competitive mechanism and iodophenyl scaffold. Imatinib (0.867), included as a historical reference anchor, ranked highest overall. Under W1, three newly approved agents ranked High (≥ 0.75), six Moderate, and three Low.

Sensitivity across the three weight schemes yielded high pairwise rank concordance (Spearman’s *ρ* = 0.956–0.989), indicating stable relative ordering under plausible weight variation. By contrast, the rank-based robustness analysis comparing wToxPi rank ordering with summed endpoint rank ordering yielded Spearman’s *ρ* = 0.835, indicating substantial—but not near-identity—concordance and reflecting the greater sensitivity of unweighted rank aggregation to the DILI-saturated distribution (Table 2; Fig. 2).

**Table 2:** Weighted ToxPi scores^a^ under three weight schemes and prioritization-tier classification

Drug	W1 wToxPi	Tier W1	W2 equal	Tier W2	W3 heavy	Tier W3	Stable?
Imatinib (anchor)	0.867	High	0.840	High	0.922	High	✓
Sevabertinib	0.860	High	0.848	High	0.885	High	✓
Zongertinib	0.840	High	0.809	High	0.902	High	✓
Lazertinib ^b^	0.775	High	0.729	Moderate	0.868	High	△ W2
Taletrectinib	0.736	Moderate	0.697	Moderate	0.816	High	△ W3
Sunvozertinib	0.708	Moderate	0.636	Moderate	0.853	High	△ W3
Defactinib ^b^	0.657	Moderate	0.581	Moderate	0.810	High	△ W3
Ensartinib	0.547	Moderate	0.459	Low	0.726	Moderate	△ W2
Avutometinib ^b^	0.525	Moderate	0.468	Low	0.640	Moderate	△ W2
Vimseltinib	0.504	Moderate	0.475	Low	0.565	Moderate	△ W2
Inavolisib ^b^	0.446	Low	0.417	Low	0.506	Moderate	△ W3
Tovorafenib	0.397	Low	0.368	Low	0.455	Low	✓
Mirdametinib	0.080	Low	0.082	Low	0.076	Low	✓

^a^W1: primary (0.30/0.30/0.20/0.20). W2: equal (0.25/0.25/0.25/0.25). W3: cardiac/hepatic heavy (0.40/0.40/0.10/0.10). ✓ = same prioritization tier across all three schemes; △ = ≥ 1 tier change in at least one scheme (W2 or W3 noted). High ≥ 0.75; Moderate 0.50–0.74; Low < 0.50

^b^Combination-regimen agents: clinical plausibility mapping carries greater interpretive uncertainty (Table 3). Imatinib shown as multi-endpoint reference anchor; not ranked within primary 2024–2025 cohort. Rank-based robustness analysis comparing wToxPi rank ordering with summed endpoint rank ordering yielded Spearman *ρ* = 0.835

**Table 3:** Directional plausibility mapping: in silico wToxPi versus FDA label safety signals and pivotal trial data

Drug	wToxPi	FDA QT/cardiac	FDA hepatotox	FDA CYP3A4	Pivotal trial safety ^b^	Contextual plausibility	Ref
Imatinib (anchor)	0.867	Yes (QTc)	Yes (Black-box hepatotox)	Yes (CYP3A4/2D6)	Multiple monotherapy trials: LFT, QTc elevation	Directionally consistent (all endpoints)	[33]
Sevabertinib	0.860	Monitor QTc	Yes (ALT monitoring)	Yes (CYP3A4)	Accelerated approval; limited monotherapy safety data	Supportive alignment; data sparse	FDA label [2]
Zongertinib	0.840	Monitor QTc	Yes (LFT monitoring)	Yes (CYP3A4)	Beamion LUNG-1 (monotherapy): ≥ Gr3 LFT	Directionally consistent	[22, 35]
Lazertinib ^a^	0.775	Yes (QTc)	Yes (ALT/AST ≥ Gr3)	Moderate CYP3A4	MARIPOSA: combination with amivantamab; ALT 8%, QTc 5%	Directionally consistent; combination confounding limits attribution	[23, 36]
Taletrectinib	0.736	Monitor QTc	Yes (transaminase)	Yes (CYP3A4/2C9)	Pooled TRUST/FDA label: ≥ Gr3 ALT 9.9%; Grade 3/4 ALT 13%	Directionally consistent	[24, 37]
Sunvozertinib	0.708	Monitor QTc	Yes (LFT monitoring)	Moderate	WU-KONG6 (monotherapy): LFT elevations	Directionally consistent	[25, 38]
Defactinib ^a^	0.657	Monitor	Yes (LFT monitoring)	Moderate	FDA approval context: combination with avutometinib; LFT monitoring requirement	Supportive; combination confounding limits attribution	[26]
Ensartinib	0.547	Yes (QTc)	Yes (hepatotoxicity)	Limited	eXalt3 (monotherapy): ≥ Gr3 ALT 5%; QTc	Directionally consistent	[27, 39]
Avutometinib ^a^	0.525	Monitor	Yes (LFT monitoring)	Moderate	FDA approval context: combination with defactinib; LFT monitoring requirement	Supportive; combination confounding limits attribution	[26]
Vimseltinib	0.504	Limited	Yes (class-level DILI)	Limited	Class-level contextual proxy: pexidartinib monotherapy (ENLIVEN)	Directionally consistent at class level; direct compound-specific clinical anchoring remains limited	[28, 40]
Inavolisib ^a,c^	0.446	Limited QT data	Yes (monitoring req’d)	Limited	INAVO120: combination with palbociclib/fulvestrant; limited QT	Partial plausibility: Low tier should not be read as low liver risk; hepatic concern is better captured by DILIcon = 1.000. Combination confounding remains substantial	[29, 41]
Tovorafenib ^c^	0.397	No QT warning	Yes (ALT ≥ Gr3)	Moderate	FIREFLY-1 (monotherapy): ALT elevations (DILI-driven)	Partial plausibility: Low tier should not be read as low liver risk; hepatic concern is better captured by DILIcon = 1.000 and monotherapy label/trial findings	[30, 34]
Mirdametinib	0.080	No QT warning	Limited DILI signal	Limited	ReNeu (monotherapy): favorable overall safety profile	Directionally consistent across all endpoints	[31, 42]

^a^Combination-regimen agents: plausibility mapping carries greater interpretive uncertainty; clinical signals may reflect additive or partner-drug pharmacology, not indexed compound alone

^b^Monotherapy trials cited unless marked as combination-regimen. Directional plausibility mapping for interpretive support only; not formal predictive validation

^c^For Inavolisib and Tovorafenib, hepatic interpretation should rely primarily on DILIcon rather than the composite tier

### Binary–continuous fusion sensitivity analysis

Comparing the primary arithmetic-mean approach with three alternative fusion scenarios showed that the principal structural findings were preserved. Under the ADMETlab-only continuous scenario, Spearman *ρ* = 0.780; DILI saturation remained unchanged (12/13 compounds ≥ 0.80), Mirdametinib remained the unique low outlier, and five compounds changed tier. The thresholded majority-vote scenario yielded Spearman *ρ* = 0.812 and again preserved the main structure, but reduced discrimination to six distinct composite values. By contrast, soft-label pseudo-probability mapping (Yes = 0.75, No = 0.25) showed near-identity concordance with the primary framework (Spearman *ρ* = 0.978), preserved 13 distinct values, and retained the 12/13 DILIcon ≥ 0.80 pattern. Across the broader soft-label grid (*p* = 0.60–0.90), rank concordance remained high (Spearman *ρ* = 0.929–0.995), and Mirdametinib remained the lowest-ranked outlier throughout. Full data are provided in Online Resource 4, Tables S4.1 A and S4.1B.

Replacing the 3-platform CYP3A4 consensus with a 2-platform alternative (ADMETlab/vNN only) produced a maximum rank displacement of 2 positions and three prioritization-tier transitions, all confined to tier-boundary compounds (e.g., Lazertinib: High → Moderate). Rank correlation remained high (Spearman *ρ* = 0.97; Kendall *τ* = 0.90), indicating localized rather than structural disruption (Online Resource 4, Table S4.2). This is mechanistically plausible because ADMETlab provides continuous probabilities, whereas vNN-ADMET and SwissADME provide binary classifications derived from different model architectures.

### Heatmap visualization

**Fig. 1:**
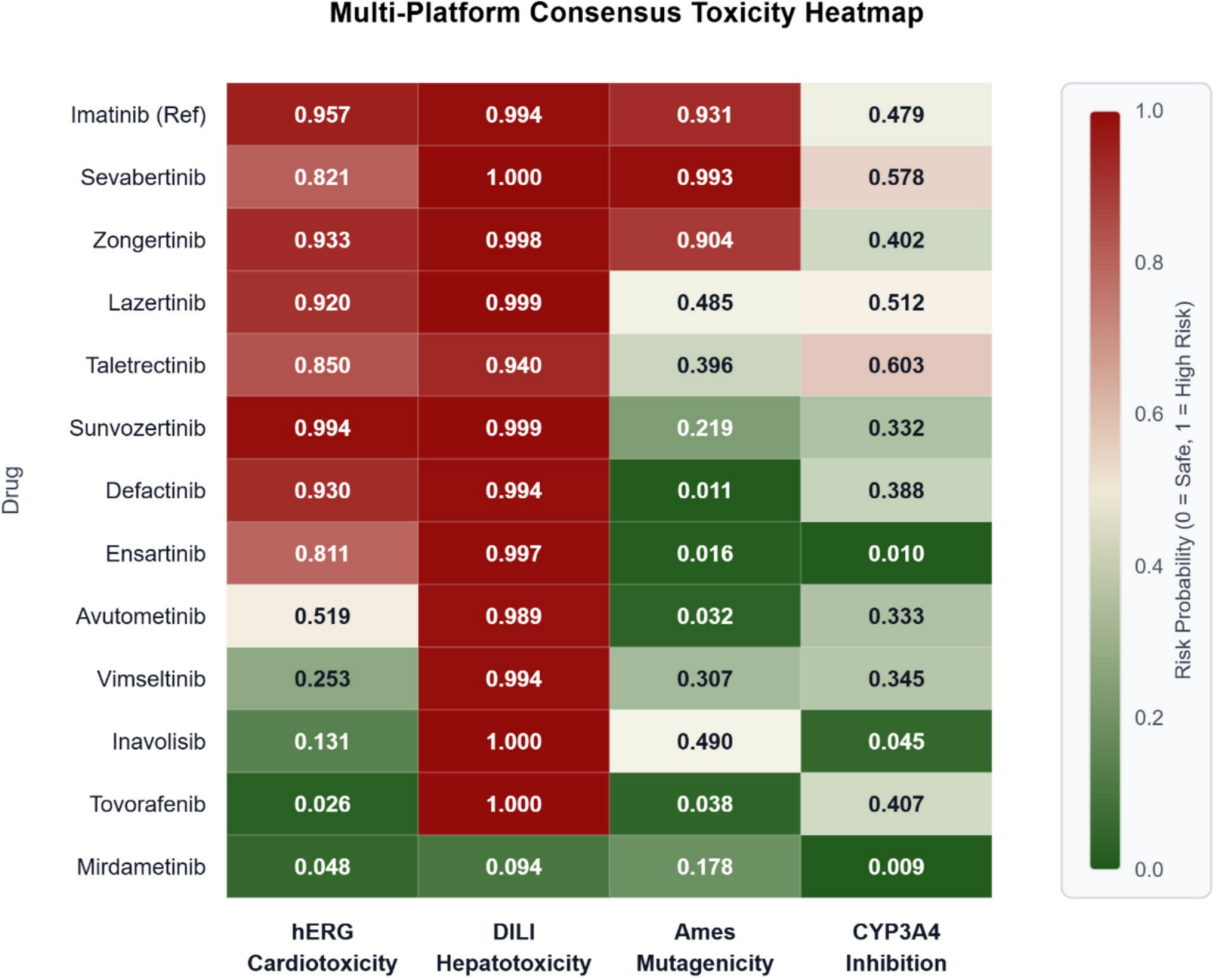
Multi-platform consensus toxicity heatmap. Four endpoint consensus scores (hERGcons, DILIcon, Amescons, CYP3A4cons) derived from ADMETlab 3.0 and vNN-ADMET (hERGcons, DILIcon, Amescons) and additionally SwissADME (CYP3A4cons only). Contextual reference data (Pred-hERG 5.0, ProTox 3.0) in Online Resource 2. Color scale: green = low predicted risk; red = high predicted risk. ‡ Mirdametinib outside Pred-hERG 5.0 applicability domain

Figure 1 highlights three class-level patterns: (1) near-universal DILI saturation, with Mirdametinib as the structural outlier; (2) a strong hERG discriminatory axis; and (3) the widest bidirectional spread in Amescons. Together, these patterns indicate that composite rank differences within this cohort are driven mainly by hERG and Ames variability within a DILI-saturated class.

Table 3 presents directional plausibility mapping between in silico predictions, FDA label safety signals, and pivotal trial adverse-event data. This comparison is contextual rather than predictive validation. Exploratory FAERS review identified at least one DILI-compatible hepatic preferred term for each newly approved agent with ≥ 10 total reports, providing additional post-marketing context for the class-level hepatic signal; strict QT-specific preferred terms were absent for 9 of 12 newly approved agents. Full FAERS results are provided in Online Resource 4, Table S4.4.

Interpretation is more uncertain for four agents evaluated primarily in combination regimens in pivotal trials, because observed hepatic or QT signals cannot be attributed to the indexed compound without disaggregated single-agent data. To reduce regimen-level confounding, a monotherapy-evaluable subset was also examined (Online Resource 4, Table S4.3).

Overall, 11 of 13 compounds (85%) showed at least one endpoint-level pattern that was directionally consistent with documented clinical safety observations. Two partial misalignments are particularly informative: Inavolisib (wToxPi 0.446/Low, DILIcon = 1.000) and Tovorafenib (wToxPi 0.397/Low, DILIcon = 1.000). Both illustrate a structural limitation of the composite: when hERGcons is near zero but DILI is saturated across the class, hepatic liability may be underrepresented by the integrated score. For these compounds, endpoint-specific DILIcon review is more informative than composite rank alone.

## Discussion

**Fig. 2:**
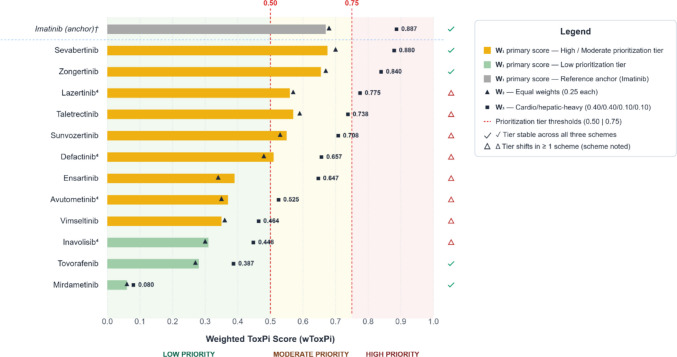
Weighted ToxPi prioritization and rank stability across three weight schemes. Bars show W1 composite scores ranked in descending order; symbols indicate corresponding W2 and W3 values. Dashed lines mark prioritization-tier thresholds, and the right-hand column shows whether tier assignment remained stable across schemes. Imatinib is shown as a historical reference anchor and was not ranked within the primary 2024–2025 cohort. Combination-regimen agents represent single-compound in silico profiling only

This framework is best understood as a transparent, hypothesis-generating prioritization tool rather than a calibrated predictor of clinical risk. Within that scope, the dominant result of the present analysis is the near-universal hepatotoxicity signal saturation observed across the newly approved kinase inhibitor cohort, with 12 of 13 compounds showing DILIcon ≥ 0.80. This finding changes the interpretive role of DILI within the present class: rather than functioning primarily as a ranking variable, it behaves more plausibly as a class-level pharmacovigilance alert. In practical terms, the composite ordering in this cohort is driven less by differences in predicted hepatotoxicity than by the wider discriminatory spread of hERG- and Ames-related signals. It is also important to acknowledge that this near-universal DILI saturation, observed across structurally and mechanistically diverse kinase inhibitors, may partly reflect a structural feature of general-purpose prediction platforms trained on broad chemical spaces. Such models may assign elevated DILI probabilities to compounds sharing the lipophilic and high-molecular-weight features common to many kinase inhibitors, even when compound-specific hepatotoxic mechanisms differ. This does not invalidate the finding as a class-level pharmacovigilance alert. The consistent elevation across agents with distinct targets and scaffolds is itself mechanistically plausible given known kinase inhibitor hepatotoxicity risk factors [21]. In this DILI-saturated kinase inhibitor cohort, wToxPi is more appropriately interpreted as a multi-endpoint prioritization framework than as a liver-specific ranking tool. Its value lies in capturing incremental differentiation from hERG-, Ames-, and CYP3A4-related liabilities against a background of class-level hepatic hazard; accordingly, a low overall wToxPi should not be interpreted as evidence of low hepatic risk, and endpoint-specific DILIcon review remains essential regardless of composite rank (Fig. 2).

The supplementary sensitivity analyses strengthen this interpretation by showing that the principal prioritization structure is more stable than any single fusion choice. When binary platform contributions were removed entirely, the internal ordering shifted but the main cohort-level findings remained intact (Online Resource 4, Table S4.1A). When the framework was forced into a more fully discretized decision structure, discrimination narrowed substantially (Online Resource 4, Table S4.1A). By contrast, soft-label pseudo-probability mapping showed near-complete concordance with the primary framework while preserving full score separation across compounds, and this pattern remained stable across the broader sensitivity grid (Online Resource 4, Tables S4.1A and S4.1B). Taken together, these findings suggest that step-function effects introduced by binary coding are statistically measurable but structurally bounded, and that the main conclusions of the study do not depend on one arbitrary fusion rule alone. The same general pattern was observed in the CYP3A4 platform-asymmetry analysis, which preserved the principal prioritization extremes despite localized boundary-level shifts (Online Resource 4, Table S4.2).

Among the newly approved agents, Sevabertinib and Zongertinib consistently occupied the highest composite ranks. For Zongertinib, this positioning is directionally strengthened by monotherapy evidence from Beamion LUNG-1 [22], whereas for Sevabertinib the post-approval clinical evidence base remains comparatively sparse [2]. More broadly, the prominence of these HER2-directed agents within the integrated ranking is mechanistically plausible in light of previously reported cardio-hepatic concerns across the broader anti-HER2 therapeutic landscape [32]. Even so, the present framework supports directional plausibility rather than predictive calibration: it identifies compounds whose integrated profiles align with emerging clinical concern, but it does not estimate clinical event probability or severity.

A major interpretive boundary concerns combination-regimen confounding. Four agents—Lazertinib, Avutometinib, Defactinib, and Inavolisib—were approved in or primarily evaluated through combination-based settings, precluding clean attribution of observed hepatic or cardiac safety patterns to the indexed kinase inhibitor alone [23, 26, 29]. The present framework should therefore be interpreted as a drug-intrinsic, single-compound prioritization tool. Combination confounding weakens direct plausibility mapping for these agents but does not invalidate the in silico scoring itself. The monotherapy-only sensitivity analysis is important in this context because it shows that the principal ranking extremes remain preserved even after these combination-based approvals are excluded from within-subset interpretation (Online Resource 4, Table S4.3).

The compounds that most clearly expose the structural limits of the composite are Inavolisib and Tovorafenib. Both carry DILIcon = 1.000 yet have low overall wToxPi ranks because hERG contribution remains minimal. For Tovorafenib, the transaminase elevations reported in FIREFLY-1 [30] are directionally consistent with the endpoint-level hepatic signal despite the low composite position. These examples illustrate an important boundary of interpretation: when one endpoint is near-saturated across the class and another varies widely, endpoint-level inspection becomes more informative than the composite rank alone. In such cases, the framework is most useful as a structured prioritization aid rather than as a substitute for endpoint-specific clinical judgment. For Inavolisib and Tovorafenib specifically, the composite prioritization tier should not be used to infer low hepatic concern. In these compounds, the Low composite rank is a consequence of near-zero hERGcons in a class where DILI is already near-saturated; it does not reflect a low hepatic liability estimate. Endpoint-specific DILIcon interpretation—supported by FDA label–based monitoring requirements and trial-level transaminase data—is the more appropriate basis for hepatic safety assessment in these two agents.

Mirdametinib remained the clearest structural outlier across all analyses. Its non-ATP-competitive MEK1/2 mechanism and iodophenyl-containing scaffold may contribute to its separation from the broader kinase inhibitor pattern [21], and its low composite profile is broadly consistent with the manageable safety experience reported in ReNeu [31]. At the same time, Mirdametinib falls outside the Pred-hERG 5.0 applicability domain; low prioritization should therefore be read as relative, not as evidence of absence of risk. It is also important to note that contextual reference platforms did not always directionally align with the formula-derived consensus scores. In selected compounds, Pred-hERG 5.0 non-blocker classifications coexisted with relatively high hERGcons values, and ProTox 3.0 inactive DILI predictions coexisted with high DILIcon values. These discrepancies do not represent internal inconsistency of the framework, because Pred-hERG 5.0 and ProTox 3.0 were intentionally excluded from formula construction and retained only for contextual triangulation across non-harmonized model architectures. Because this applicability-domain caveat affects the hERG-related interpretation of Mirdametinib most directly, its low-tier ranking warrants greater caution than compounds whose predictions fall fully within established model boundaries.

The exploratory FAERS analysis adds a translationally relevant but deliberately limited post-marketing contextual layer (Online Resource 4, Table S4.4). At least one DILI-compatible hepatic preferred term was identified for every newly approved agent with ≥ 10 total reports, whereas strict QT-specific preferred terms were absent for 9 of 12 newly approved agents, a pattern directionally consistent with class-level DILIcon saturation and variable hERGcons discrimination. Although FAERS cannot serve as formal validation here, its value is supportive rather than confirmatory. Importantly, because exposure windows range from approximately 5 months for Sevabertinib (approved November 2025) to nearly 2 years for Tovorafenib (approved April 2024), report counts are not directly comparable between compounds and are interpreted only as the presence or absence of supportive contextual reporting; a low count in a recently approved agent reflects limited exposure time rather than lower risk.

Taken together, the present analysis supports a balanced conclusion. The framework appears most useful for identifying relative multi-domain safety-priority patterns within a newly approved kinase inhibitor cohort, especially in a setting where class-level hepatotoxicity is widespread and hERG/Ames variability provides additional discriminatory structure. Its principal strengths are transparency, reproducibility, and robustness at the level of broad prioritization patterns. Its principal limitations are equally clear: heterogeneous platform outputs, heuristic weighting, endpoint asymmetry, immature post-marketing context, and constrained clinical attribution for combination-based approvals. Framed in this way, the work is not a surrogate for clinical safety prediction, but it does provide a rational starting point for pharmacovigilance-oriented hypothesis generation and for prioritizing which agents warrant closer endpoint-specific scrutiny in early post-approval use. A further interpretive boundary concerns the imatinib anchor: because imatinib occupies the highest composite rank in the full dataset, a Moderate-tier newly approved agent should not be interpreted as clinically safer than imatinib in an absolute sense. The prioritization tiers are relative labels within the present analytic framework, and imatinib was included as a historical contextual anchor rather than as a safety ceiling.

## Conclusion

Multi-platform in silico consensus modeling and weighted ToxPi integration provide a transparent, reproducible framework for pharmacovigilance-oriented prioritization of newly approved KIs. The dominant finding—near-universal DILIcon ≥ 0.80 across 12 of 13 compounds—identifies hepatotoxicity as a class pharmacovigilance imperative rather than an inter-compound discriminator, with composite ranking driven mainly by hERGcons and Amescons variability. Within the 2024–2025 cohort, Sevabertinib, Zongertinib, and Lazertinib occupied the highest composite ranks, whereas Mirdametinib remained the structural outlier consistent with its distinct mechanism and scaffold. The principal findings were robust across systematic sensitivity analyses addressing binary–continuous fusion alternatives, CYP3A4 platform asymmetry, and combination-regimen confounding, confirming that the main conclusions do not depend on any single analytical choice. Directional plausibility with FDA label safety data was observed for most compounds, and exploratory FAERS review provided early post-marketing support for the class-level hepatic signal. Combination-regimen confounding increases interpretive uncertainty for four agents and limits clean regimen-level validation of this single-compound framework. For Inavolisib and Tovorafenib, the composite underrepresents hepatic liability despite DILIcon = 1.000, and endpoint-specific transaminase surveillance remains warranted irrespective of composite rank. Future work should integrate free plasma Cmax exposure data with wToxPi endpoint scores, apply confidence-weighted fusion for heterogeneous platform outputs, and extend pharmacovigilance linkage to prospective registry data and systematic signal detection algorithms.

## Supplementary Information

Below is the link to the electronic supplementary material.

ESM 1 (PDF 160 KB)

ESM 2 (PDF 207 KB)

ESM 3 (PDF 185 KB)

ESM 4 (PDF 273 KB)

## Data Availability

All SMILES strings used as platform inputs are provided in Online Resource 3 (Table S3.2). All raw platform outputs and consensus scores are derived in Online Resource 3 (Table S3.1). ProTox 3.0 output is in Online Resource 2. Pred-hERG 5.0 output is in Online Resource 2. The complete wToxPi calculation dataset is available from the corresponding author on reasonable request. All five prediction platforms are publicly accessible.
