# Supplementary material for: Class-level DILI saturation and hERG-driven risk stratification among 2024–2025 FDA-approved kinase inhibitors: an in silico multi-platform safety analysis
Source: Invest New Drugs. 2026 May 2;44(3):253–63. doi: 10.1007/s10637-026-01615-1 (PMC13486050; doi:10.1007/s10637-026-01615-1)
Supplement: Supplementary file 1 [file 10637_2026_1615_MOESM1_ESM.pdf]

Online Resource 1

Class-Level DILI Saturation and hERG-Driven Risk Stratification Among 2024–2025 FDA-Approved Kinase Inhibitors: An In Silico Multi-Platform Safety Analysis

Journal: Investigational New Drugs (Springer)

Authors: Nihan Küçük Yılmaz[1], Veysel Baskın[2]

[1]Yalova University, Faculty of Medicine, Dept. Medical Pharmacology, Yalova, Turkey

[2]Hitit University, Faculty of Medicine, Dept. Medical Pharmacology, Çorum, Turkey

*Corresponding author: nihan.kucuk@yalova.edu.tr

Online Resource 1: Step-by-step worked example of wToxPi calculation for Sevabertinib, with all platform inputs, consensus score derivation steps, and final composite score.

Worked Example: Step-by-Step wToxPi Calculation for Sevabertinib

This worked example demonstrates the complete consensus scoring and wToxPi calculation for Sevabertinib using the W1 primary weight scheme. All raw platform inputs are shown to ensure full reproducibility.

Step 1 — Raw platform inputs

ADMETlab 3.0 (continuous, 0–1):

hERG = 0.642 | DILI = 1.000 | Ames = 0.985 | CYP3A4-inh = 0.734

vNN-ADMET (binary Yes/No → coded 1/0):

hERG Blocker = Yes →1 | DILI = Yes →1 | Ames = Yes →1 | CYP3A4 Inhibitor = No →0

SwissADME (categorical Yes/No → coded 1/0):

CYP3A4 Inhibitor = Yes →1

Pred-hERG 5.0 (contextual reference only — NOT used in formula):

Prediction = Blocker | Confidence = 51.79% | Applicability Domain = Inside | Potency = Moderate blocker | IC_{50} = 5.42 μM

Step 2 — Consensus score derivation

hERGcons = (ADMETlab_hERG + vNN_hERG) / 2 = (0.642 + 1) / 2 = 0.821

DILIcon = (ADMETlab_DILI + vNN_DILI) / 2 = (1.000 + 1) / 2 = 1.000

Amescons = (ADMETlab_Ames + vNN_Ames) / 2 = (0.985 + 1) / 2 = 0.993

CYP3A4cons = (ADMETlab_CYP3A4 + vNN_CYP3A4 + Swiss_CYP3A4) / 3 = (0.734 + 0 + 1) / 3 = 0.578

Note on binary fusion (Ames): vNN_Ames = Yes → 1.0 (binary). Even if ADMETlab_Ames were lower, the binary "Yes" would contribute exactly 0.5 to the two-platform consensus. This step-function effect is discussed in the Limitations section of the main manuscript.

Step 3 — wToxPi calculation (W1 primary scheme: 0.30/0.30/0.20/0.20)

wToxPi = (0.30 × hERGcons) + (0.30 × DILIcon) + (0.20 × Amescons) + (0.20 × CYP3A4cons)

wToxPi = (0.30 × 0.821) + (0.30 × 1.000) + (0.20 × 0.993) + (0.20 × 0.578)

wToxPi = 0.2463 + 0.3000 + 0.1986 + 0.1156

wToxPi = 0.860 ✓ → Prioritization Tier: HIGH (≥0.75)

Step 4 — Sensitivity analysis (W2 and W3)

W2 equal (0.25/0.25/0.25/0.25):

$wToxPi_2$ = (0.25×0.821) + (0.25×1.000) + (0.25×0.993) + (0.25×0.578) = 0.848 → HIGH

W3 cardiac/hepatic heavy (0.40/0.40/0.10/0.10):

$wToxPi_3$ = (0.40×0.821) + (0.40×1.000) + (0.10×0.993) + (0.10×0.578) = 0.885 → HIGH

Rank stability: same band (High) across all three weight schemes ✓

Full raw data for all 13 compounds are provided in Online Resource 3 (Table S3.1).
