# Supplementary material for: Class-level DILI saturation and hERG-driven risk stratification among 2024–2025 FDA-approved kinase inhibitors: an in silico multi-platform safety analysis
Source: Invest New Drugs. 2026 May 2;44(3):253–63. doi: 10.1007/s10637-026-01615-1 (PMC13486050; doi:10.1007/s10637-026-01615-1)
Supplement: Supplementary file 2 [file 10637_2026_1615_MOESM2_ESM.pdf]

Online Resource 2

Class-Level DILI Saturation and hERG-Driven Risk Stratification Among 2024–2025 FDA-Approved Kinase Inhibitors: An In Silico Multi-Platform Safety Analysis

Journal: Investigational New Drugs (Springer)

Authors: Nihan Küçük Yılmaz[1], Veysel Baskın[2]

[1]Yalova University, Faculty of Medicine, Dept. Medical Pharmacology, Yalova, Turkey

[2]Hitit University, Faculty of Medicine, Dept. Medical Pharmacology, Çorum, Turkey

*Corresponding author: nihan.kucuk@yalova.edu.tr

Online Resource 2: Physicochemical properties (MW, LogP, TPSA, HBD, HBA, QED, Lipinski Ro5 [19], GI absorption and full ProTox 3.0 and Pred-hERG 5.0 contextual reference output tables for all 13 study compounds.

Table S2.1 Physicochemical properties and drug-likeness descriptors for all 13 study compounds (source: ADMETlab 3.0 and SwissADME; accessed March 2026)

Drug	MW (Da)	LogP	TPSA (Å²)	HBD	HBA	QED	Lipinski Ro5*	GI Abs.
Sevabertinib	484.15	2.53	106.73	3	9	0.470	Pass	High
Zongertinib†	535.24	4.72	122.98	2	11	0.292	2 violations	Low
Sunvozertinib†	584.01	4.44	114.88	4	10	0.237	1 violation	Low
Taletrectinib	405.20	4.36	77.47	3	6	0.475	Pass	High
Avutometinib	471.10	1.63	136.31	2	10	0.393	Pass	Low
Defactinib†	510.14	2.66	142.10	3	11	0.416	2 violations	Low
Vimseltinib	431.21	3.23	99.75	1	9	0.498	Pass	Low
Mirdametinib	482.00	2.61	90.82	4	6	0.360	Pass	Low
Ensartinib†	560.15	3.83	122.47	4	9	0.373	1 violation	Low
Inavolisib	407.14	0.82	111.71	3	9	0.782	Pass	Low
Lazertinib†	554.28	3.54	109.67	2	11	0.280	2 violations	Low
Tovorafenib†	505.01	2.82	135.78	4	9	0.476	1 violation	Low
Imatinib (anchor)	493.26	2.59	86.28	2	8	0.389	Pass	High

a Lipinski's Rule of Five: MW ≤500 Da, logP ≤5, HBD ≤5, and HBA ≤10. Violation counts indicate the number of thresholds exceeded. † Exceeds the 500 Da molecular-weight threshold. HBD: hydrogen bond donors; HBA: hydrogen bond acceptors; QED: quantitative estimate of drug-likeness (0–1; higher = more drug-like). GI absorption categories are reported as provided by SwissADME. In this dataset, ten compounds were classified as having predicted Low GI absorption. These platform-derived estimates should be interpreted cautiously and should not be taken as direct surrogates of clinical exposure.

Table S2.2 Full ProTox 3.0 contextual reference outputs for all 13 study compounds (source: ProTox 3.0, https://protox.h-its.org; accessed March 2026). These outputs are provided as contextual reference data only and were NOT incorporated into wToxPi formula inputs

**Drug**	**DILI**	**Cardiotoxicity**	**Mutagenicity**	**CYP3A4**	**CYP2D6**	**CYP2C9**
Sevabertinib	Inactive (0.59)	Inactive (0.61)	Inactive (0.50)	Inactive (0.57)	**Active (0.52)**	Inactive (0.61)
Zongertinib	Inactive (0.74)	Inactive (0.81)	Inactive (0.50)	Inactive (0.71)	Inactive (0.66)	Inactive (0.55)
Sunvozertinib	Inactive (0.56)	Inactive (0.77)	Inactive (0.69)	Inactive (0.64)	Inactive (0.65)	Inactive (0.53)
Taletrectinib	**Active (0.52)**	Inactive (0.79)	Inactive (0.51)	**Active (0.50)**	**Active (0.53)**	Inactive (0.52)
Avutometinib	**Active (0.60)**	Inactive (0.63)	Inactive (0.57)	Inactive (0.61)	Inactive (0.72)	**Active (0.60)**
Defactinib	Inactive (0.59)	Inactive (0.85)	Inactive (0.70)	Inactive (0.58)	Inactive (0.62)	Inactive (0.61)
Vimseltinib	**Active (0.53)**	Inactive (0.85)	Inactive (0.56)	Inactive (0.79)	Inactive (0.86)	Inactive (0.66)
Mirdametinib	Inactive (0.69)	**Active (0.52)**	**Active (0.55)**	Inactive (0.68)	Inactive (0.68)	Inactive (0.66)
Ensartinib	Inactive (0.53)	Inactive (0.79)	Inactive (0.54)	Inactive (0.65)	Inactive (0.58)	**Active (0.53)**
Inavolisib	Inactive (0.60)	Inactive (0.82)	Inactive (0.66)	Inactive (0.78)	Inactive (0.73)	Inactive (0.75)
Lazertinib	Inactive (0.57)	Inactive (0.76)	Inactive (0.55)	Inactive (0.70)	Inactive (0.71)	Inactive (0.63)
Tovorafenib	**Active (0.66)**	Inactive (0.90)	Inactive (0.56)	Inactive (0.72)	Inactive (0.63)	Inactive (0.61)
Imatinib (anchor)	**Active (0.71)**	Inactive (0.84)	Inactive (0.73)	Inactive (0.72)	**Active (0.63)**	Inactive (0.79)

[a]Active predictions (bold, dark red) indicate the compound is predicted to exhibit the respective endpoint; Inactive = predicted negative. Probability values in parentheses (0–1; higher = greater model confidence in Active). ProTox 3.0 outputs are contextual reference only; not used in consensus formula. All 13 compounds within ProTox 3.0 applicability domain

Table S2.3 Pred-hERG 5.0 contextual reference outputs for all 13 study compounds (source: Pred-hERG 5.0; accessed March 2026). These outputs are provided as contextual reference data only and were NOT incorporated into hERGcons formula inputs

Drug	Prediction	Pred. Conf.	App. Domain	Potency Class	Potency Conf.	pIC_{50}
Sevabertinib	**Blocker**	51.79%	Inside	Moderate blocker	33.65%	5.420
Zongertinib	**Blocker**	70.91%	Inside	Moderate blocker	31.80%	5.426
Sunvozertinib	**Blocker**	60.61%	Inside	Moderate blocker	37.10%	5.208
Taletrectinib	**Blocker**	88.21%	Inside	Moderate blocker	36.70%	5.362
Avutometinib	Non-blocker	73.70%	Inside	Weak-blocker	30.20%	5.272
Defactinib	**Blocker**	63.64%	Inside	Moderate blocker	34.00%	5.282
Vimseltinib	Non-blocker	75.07%	Inside	Moderate blocker	36.90%	5.386
Mirdametinib ‡	Non-blocker	89.06%	Outside ‡	Weak-blocker	38.02%	5.307
Ensartinib	Non-blocker	54.67%	Inside	Moderate blocker	36.05%	5.226
Inavolisib	Non-blocker	97.95%	Inside	Moderate blocker	34.67%	5.338
Lazertinib	Non-blocker	55.46%	Inside	Weak-blocker	32.80%	5.281
Tovorafenib	Non-blocker	96.94%	Inside	Weak-blocker	33.00%	5.152
Imatinib (anchor)	**Blocker**	74.38%	Inside	Moderate blocker	36.18%	5.472

‡ Mirdametinib falls outside Pred-hERG 5.0 applicability domain; its hERGcons score (0.048) derived from ADMETlab 3.0 and vNN-ADMET should be interpreted with lower confidence for this compound. Pred. Conf.: prediction confidence (%); App. Domain: applicability domain status; Potency Conf.: potency class confidence (%); pIC_{50}: predicted hERG inhibitory potency. Blocker = predicted hERG channel blocker; Non-blocker = predicted non-blocker
