# Supplementary material for: Class-level DILI saturation and hERG-driven risk stratification among 2024–2025 FDA-approved kinase inhibitors: an in silico multi-platform safety analysis
Source: Invest New Drugs. 2026 May 2;44(3):253–63. doi: 10.1007/s10637-026-01615-1 (PMC13486050; doi:10.1007/s10637-026-01615-1)
Supplement: Supplementary file 3 [file 10637_2026_1615_MOESM3_ESM.pdf]

Online Resource 3

Class-Level DILI Saturation and hERG-Driven Risk Stratification Among 2024–2025 FDA-Approved Kinase Inhibitors: An In Silico Multi-Platform Safety Analysis

Journal: Investigational New Drugs (Springer)

Authors: Nihan Küçük Yılmaz[1], Veysel Baskın[2]

[1]Yalova University, Faculty of Medicine, Dept. Medical Pharmacology, Yalova, Turkey

[2]Hitit University, Faculty of Medicine, Dept. Medical Pharmacology, Çorum, Turkey

*Corresponding author: nihan.kucuk@yalova.edu.tr

Online Resource 3: Complete reproducibility package containing Table S3.1 (all raw platform-level outputs and derived consensus scores), Table S3.2 (canonical SMILES strings for all 13 study compounds), and Table S3.3 (endpoint-level platform outputs, native scales, and 0–1 coding used for consensus derivation and wToxPi formula).

Table S3.1 All raw platform-level outputs and derived endpoint consensus scores for all 13 study compounds (formula inputs). ADMETlab 3.0 values from continuous probability scores; vNN-ADMET and SwissADME binary outputs coded Yes=1/No=0. Bold columns = consensus scores entered into wToxPi formula

Drug	hERG			DILI			Ames			CYP3A4			
	ADMET	vNN	cons	ADMET	vNN	cons	ADMET	vNN	cons	ADMET	vNN	Swiss	cons
Sevabertinib	0.642	1	**0.821**	1.000	1	**1.000**	0.985	1	**0.993**	0.734	0	1	**0.578**
Zongertinib	0.865	1	**0.933**	0.996	1	**0.998**	0.808	1	**0.904**	0.205	0	1	**0.402**
Sunvozertinib	0.987	1	**0.994**	0.997	1	**0.999**	0.437	0	**0.219**	0.997	0	0	**0.332**
Taletrectinib	0.699	1	**0.850**	0.879	1	**0.940**	0.791	0	**0.396**	0.808	0	1	**0.603**
Avutometinib	0.037	1	**0.519**	0.977	1	**0.989**	0.064	0	**0.032**	0.000	0	1	**0.333**
Defactinib	0.860	1	**0.930**	0.988	1	**0.994**	0.021	0	**0.011**	0.165	0	1	**0.388**
Vimseltinib	0.505	0	**0.253**	0.988	1	**0.994**	0.613	0	**0.307**	0.036	0	1	**0.345**
Mirdametinib ‡	0.096	0	**0.048**	0.187	0	**0.094**	0.355	0	**0.178**	0.027	0	0	**0.009**
Ensartinib	0.621	1	**0.811**	0.994	1	**0.997**	0.032	0	**0.016**	0.030	0	0	**0.010**
Inavolisib	0.262	0	**0.131**	0.999	1	**1.000**	0.980	0	**0.490**	0.134	0	0	**0.045**
Lazertinib	0.840	1	**0.920**	0.998	1	**0.999**	0.970	0	**0.485**	0.536	0	1	**0.512**
Tovorafenib	0.052	0	**0.026**	0.999	1	**1.000**	0.076	0	**0.038**	0.222	0	1	**0.407**
Imatinib (anchor)	0.913	1	**0.957**	0.988	1	**0.994**	0.862	1	**0.931**	0.438	0	1	**0.479**

[a]hERGcons = mean(ADMETlab_hERG, vNN_hERG). DILIcon = mean(ADMETlab_DILI, vNN_DILI). Amescons = mean(ADMETlab_Ames, vNN_Ames). CYP3A4cons = mean(ADMETlab_CYP3A4, vNN_CYP3A4, Swiss_CYP3A4). ADMETlab: continuous 0–1; vNN-ADMET/SwissADME: binary 1=Yes/0=No. ‡ Mirdametinib outside Pred-hERG 5.0 applicability domain; hERGcons for this compound should be interpreted with lower confidence. Pred-hERG 5.0 outputs are in Table S2.3 (Online Resource 2); not used in any formula.

Table S3.2 Canonical SMILES strings for all 13 study compounds as submitted to all five prediction platforms (source: PubChem; accessed March 2026)

Drug	Canonical SMILES (PubChem; accessed March 2026)
Sevabertinib	COC1=C(C=CC=C1Cl)NC2=C(NC3=C2C(=O)NCC3)C4=C(C=NC=C4)OC[C@@H]5COCCO5
Zongertinib	CC1=C(C=CC(=C1)NC2=NC=NC3=CN=C(N=C32)N4CCC(CC4)NC(=O)C=C)OC5=CC6=C(C=C5)N(C=N6)C
Sunvozertinib	CC(C)(C1=CC(=C(C=C1NC2=NC(=NC=C2)NC3=C(C=C(C(=C3)NC(=O)C=C)N4CC[C@H](C4)N(C)C)OC)Cl)F)O
Taletrectinib	C[C@H](COC1=CC=C(C=C1)C2=CN=C3N2N=C(C=C3)N[C@H](C)C4=CC(=CC=C4)F)N
Avutometinib	CC1=C(C(=O)OC2=C1C=CC(=C2)OC3=NC=CC=N3)CC4=C(C(=NC=C4)NS(=O)(=O)NC)F
Defactinib	CNC(=O)C1=CC=C(C=C1)NC2=NC=C(C(=N2)NCC3=NC=CN=C3N(C)S(=O)(=O)C)C(F)(F)F
Vimseltinib	CC1=C(C=CC(=N1)C2=CN=C(N(C2=O)C)NC(C)C)OC3=CC(=NC=C3)C4=CN(N=C4)C
Mirdametinib	C1=CC(=C(C=C1I)F)NC2=C(C=CC(=C2F)F)C(=O)NOC[C@@H](CO)O
Ensartinib	C[C@@H]1CN(C[C@@H](N1)C)C(=O)C2=CC=C(C=C2)NC(=O)C3=NN=C(C(=C3)O[C@H](C)C4=C(C=CC(=C4Cl)F)Cl)N
Inavolisib	C[C@@H](C(=O)N)NC1=CC2=C(C=C1)C3=NC(=CN3CCO2)N4[C@@H](COC4=O)C(F)F
Lazertinib	CN(C)CC1=CN(N=C1C2=CC=CC=C2)C3=NC(=NC=C3)NC4=C(C=C(C(=C4)NC(=O)C=C)N5CCOCC5)OC
Tovorafenib	C[C@H](C1=NC=C(S1)C(=O)NC2=NC=C(C(=C2)C(F)(F)F)Cl)NC(=O)C3=C(C(=NC=N3)N)Cl
Imatinib (anchor)	CC1=C(C=C(C=C1)NC(=O)C2=CC=C(C=C2)CN3CCN(CC3)C)NC4=NC=CC(=N4)C5=CN=CC=C5

[a]SMILES strings were used as inputs to ADMETlab 3.0, vNN-ADMET, Pred-hERG 5.0, ProTox 3.0, and SwissADME. Canonical SMILES from PubChem; stereochemistry preserved where specified

Table S3.3 Endpoint/platform/coding reference for consensus score derivation and wToxPi formula

Endpoint	Formula platforms	Native output(s)	0-1 coding / fusion rule	Used in formula?	Notes
hERGcons	ADMETlab 3.0; vNN-ADMET	ADMETlab: continuous probability (0-1); vNN: Yes/No	vNN Yes=1, No=0; hERGcons = mean(ADMETlab_hERG, vNN_hERG)	Yes	Pred-hERG 5.0 is contextual reference only and is not included in the formula.
DILIcon	ADMETlab 3.0; vNN-ADMET	ADMETlab: continuous probability (0-1); vNN: Yes/No	vNN Yes=1, No=0; DILIcon = mean(ADMETlab_DILI, vNN_DILI)	Yes	ProTox 3.0 DILI output is contextual reference only and is not included in the formula.
Amescons	ADMETlab 3.0; vNN-ADMET	ADMETlab: continuous probability (0-1); vNN: Yes/No	vNN Yes=1, No=0; Amescons = mean(ADMETlab_Ames, vNN_Ames)	Yes	No additional contextual platform was included in the formula for Ames.
CYP3A4cons	ADMETlab 3.0; vNN-ADMET; SwissADME	ADMETlab: continuous probability (0-1); vNN: Yes/No; SwissADME: Yes/No	vNN/SwissADME Yes=1, No=0; CYP3A4cons = mean(ADMETlab_CYP3A4, vNN_CYP3A4, Swiss_CYP3A4)	Yes	SwissADME was queried for CYP3A4 inhibitor prediction only; hERG and DILI endpoints are not reported by this platform.
Pred-hERG 5.0	Pred-hERG 5.0	Categorical blocker/non-blocker; potency class; IC50 estimate	Not harmonized to 0-1; excluded from formula	No	Used only for contextual triangulation because outputs are not directly comparable to the consensus inputs.
ProTox 3.0	ProTox 3.0	Categorical/organ-toxicity predictions with confidence scores	Not harmonized to 0-1; excluded from formula	No	Used only for contextual triangulation; not entered into consensus derivation or wToxPi computation.

[a]Because hERGcons, DILIcon, and Amescons each average one continuous ADMETlab output with one binary vNN output, a binary "Yes" contributes exactly 0.5 to the two-platform consensus regardless of the underlying continuous value. This may induce step-function effects, variance compression, and reduced discrimination between compounds when continuous probabilities differ meaningfully.[b] wToxPi formula (W1 primary): 0.30×hERGcons + 0.30×DILIcon + 0.20×Amescons + 0.20×CYP3A4cons. W2 equal: 0.25/0.25/0.25/0.25. W3 cardiac/hepatic heavy: 0.40/0.40/0.10/0.10. Prioritization tiers (heuristic, a priori): High ≥0.75; Moderate 0.50–0.74; Low <0.50. Arithmetic-mean fusion of heterogeneous outputs is a pragmatic heuristic; see Limitations in main manuscript.
