# Supplementary material for: Class-level DILI saturation and hERG-driven risk stratification among 2024–2025 FDA-approved kinase inhibitors: an in silico multi-platform safety analysis
Source: Invest New Drugs. 2026 May 2;44(3):253–63. doi: 10.1007/s10637-026-01615-1 (PMC13486050; doi:10.1007/s10637-026-01615-1)
Supplement: Supplementary file 4 [file 10637_2026_1615_MOESM4_ESM.pdf]

Online Resource 4

Sensitivity Analyses and Exploratory FAERS Context

Class-Level DILI Saturation and hERG-Driven Risk Stratification Among 2024–2025 FDA-Approved Kinase Inhibitors: An In Silico Multi-Platform Safety Analysis

Journal: Investigational New Drugs (Springer)

Authors: Nihan Küçük Yılmaz[1], Veysel Baskın[2]

Online Resource 4: Additional robustness analyses and exploratory post-marketing context, including: (A) compound-level alternative fusion sensitivity analysis comparing the primary arithmetic-mean framework with ADMETlab-only continuous, thresholded majority-vote, and soft-label pseudo-probability mapping scenarios (Table S4.1A); (B) soft-label pseudo-probability sensitivity grid across p = 0.60–0.90 (Table S4.1B); (C) CYP3A4 platform-asymmetry sensitivity analysis comparing 3-platform and 2-platform consensus scenarios (Table S4.2); (D) monotherapy-evaluable sensitivity analysis excluding combination-regimen agents, with the historical Imatinib anchor excluded from within-subset rank interpretation (Table S4.3); and (E) exploratory FAERS-based post-marketing contextual summary aligned to the ToxPi order (Table S4.4).

Table S4.1A. Compound-level sensitivity analysis of binary-continuous fusion and alternative recoding scenarios. Current arithmetic-mean framework, ADMETlab-only continuous reference, soft-label pseudo-probability mapping (Yes = 0.75, No = 0.25), and thresholded majority-vote scenario

Drug	Current fusion wToxPi	ADMETlab-only wToxPi	ΔwToxPi (ADMET-only - current)	Rank current	Rank ADMET-only	Rank shift	Tier current	Tier ADMET-only	Soft-label wToxPi	Rank (SL)	Tier (SL)	Majority-vote wToxPi	Rank (MV)	Tier (MV)
Imatinib	0.867	0.830	-0.037	1	4	+3	High	High	0.767	1	High	0.800	4	High
Sevabertinib	0.860	0.836	-0.024	2	3	+1	High	High	0.760	2	High	1.000	1	High
Zongertinib	0.840	0.761	-0.079	3	6	+3	High	High	0.740	3	Moderate	0.800	4	High
Lazertinib	0.775	0.853	+0.078	4	2	-2	High	High	0.725	4	Moderate	1.000	1	High
Taletrectinib	0.736	0.793	+0.057	5	5	+0	Moderate	High	0.686	6	Moderate	1.000	1	High
Sunvozertinib	0.708	0.882	+0.174	6	1	-5	Moderate	High	0.691	5	Moderate	0.600	7	Moderate
Defactinib	0.657	0.592	-0.065	7	8	+1	Moderate	Moderate	0.607	7	Moderate	0.600	7	Moderate
Ensartinib	0.547	0.497	-0.051	8	10	+2	Moderate	Low	0.531	8	Moderate	0.600	7	Moderate
Avutometinib	0.525	0.317	-0.208	9	12	+3	Moderate	Low	0.475	11	Low	0.600	7	Moderate
Vimseltinib	0.504	0.578	+0.073	10	9	-1	Moderate	Moderate	0.529	9	Moderate	0.800	4	High
Inavolisib	0.446	0.601	+0.155	11	7	-4	Low	Moderate	0.504	10	Moderate	0.500	11	Moderate
Tovorafenib	0.397	0.375	-0.022	12	11	-1	Low	Low	0.422	12	Low	0.300	12	Low
Mirdametinib	0.080	0.161	+0.082	13	13	+0	Low	Low	0.213	13	Low	0.000	13	Low

[a]Current fusion = arithmetic-mean integration of continuous ADMETlab probabilities with binary vNN-ADMET/SwissADME outputs. ADMETlab-only = continuous-only alternative. Tier cutoffs: High >= 0.75; Moderate 0.50 - 0.74; Low < 0.50. Soft-label (SL) scenario: binary vNN-ADMET and SwissADME outputs recoded using soft-label pseudo-probability mapping (Yes = 0.75, No = 0.25), responsive to the reviewer's suggestion to explore pseudo-probability recoding of binary outputs; arithmetic-mean fusion was then applied to the resulting pseudo-continuous values. Spearman rho = 0.978 between the current framework and the soft-label scenario; 13 distinct composite values were preserved. Majority-vote (MV) scenario: ADMETlab 3.0 outputs binarized at a 0.5 threshold; 2-platform endpoints were scored positive if >=1 platform voted positive, and 3-platform CYP3A4 was scored positive if >=2 platforms voted positive. Across the alternative scenarios shown here, class-level DILI saturation remained preserved and Mirdametinib remained the unique low-scoring outlier. The principal upper-ranking structure was preserved under soft-label pseudo-probability mapping, whereas the majority-vote scenario yielded a visibly more discretized score structure.

Table S4.1B. Soft-label pseudo-probability sensitivity grid: rank concordance and structural stability across alternative Yes/No assignments

Scenario summary for pseudo-probability recording of binary outputs (p = Yes; q = 1-p for No)

p (Yes)	q (No)	Spearman ρ vs current	Tier transitions	DILI >= 0.80	Mirdametinib rank
0.60	0.40	0.929	6	1/13	Yes
0.65	0.35	0.945	6	11/13	Yes
0.70	0.30	0.967	6	11/13	Yes
**0.75**	**0.25**	**0.978**	**4**	**12/13**	**Yes**
0.80	0.20	0.984	2	12/13	Yes
0.85	0.15	0.995	2	12/13	Yes
0.90	0.10	0.995	0	12/13	Yes

[a] p = pseudo-probability assigned to binary Yes outputs; q = 1-p assigned to No outputs. Spearman rho was computed between the primary arithmetic-mean framework and each soft-label pseudo-probability mapping scenario. Tier transitions = number of compounds with tier change versus the primary scenario. DILI >= 0.80 = number of compounds with DILIcon >= 0.80 under each scenario. The highlighted row (p = 0.75) corresponds to the primary soft-label pseudo-probability mapping scenario reported in Table S4.1A. p = 0.75 is the minimum value within this grid that preserves the 12/13 DILI-saturation pattern observed under arithmetic-mean fusion, while maintaining 13 distinct composite values and near-identity rank concordance. Mirdametinib remained rank 13 (unique low-scoring outlier) across the entire grid.

Table S4.2. CYP3A4 3-platform versus 2-platform sensitivity analysis: rank-transition and tier effects on wToxPi prioritization

Drug	CYP3A4cons (3p)	CYP3A4cons (2p)	wToxPi (3p)	wToxPi (2p)	ΔwToxPi	Rank (3p)	Rank (2p)	Rank shift	Tier (3p)	Tier (2p)
Imatinib (Ref)	0.479	0.219	0.867	0.815	+0.052	1	2	-1	High	High
Sevabertinib	0.578	0.367	0.861	0.818	+0.042	2	1	+1	High	High
Zongertinib	0.402	0.102	0.840	0.781	+0.060	3	3	0	High	High
Lazertinib	0.512	0.268	0.775	0.726	+0.049	4	5	-1	High	Moderate
Taletrectinib	0.603	0.404	0.737	0.697	+0.040	5	6	-1	Moderate	Moderate
Sunvozertinib	0.332	0.498	0.708	0.741	-0.033	6	4	+2	Moderate	Moderate
Defactinib	0.388	0.083	0.657	0.596	+0.061	7	7	0	Moderate	Moderate
Ensartinib	0.010	0.015	0.548	0.549	-0.001	8	8	0	Moderate	Moderate
Avutometinib	0.333	0.000	0.525	0.459	+0.067	9	9	0	Moderate	Low
Vimseltinib	0.345	0.018	0.505	0.439	+0.066	10	11	-1	Moderate	Low
Inavolisib	0.045	0.067	0.446	0.451	-0.004	11	10	+1	Low	Low
Tovorafenib	0.407	0.111	0.397	0.338	+0.059	12	12	0	Low	Low
Mirdametinib	0.009	0.013	0.080	0.081	-0.001	13	13	0	Low	Low

[a]3p = 3-platform CYP3A4 consensus (ADMETlab/vNN-ADMET/SwissADME); 2p = 2-platform alternative (ADMETlab/vNN-ADMET). wToxPi = 0.30×hERGcons + 0.30×DILIcon + 0.20×Amescons + 0.20×CYP3A4cons. Tier cutoffs: High >=0.75; Moderate 0.50-0.74; Low < 0.50. Spearman ρ = 0.973 and Kendall τ = 0.897 (both p< 0.0001). Maximum rank shift = 2, and the top-3 set remained unchanged.

Table S4.3. Monotherapy-only sensitivity analysis after exclusion of explicitly combination-based approvals

Retained agent	W1 wToxPi	Rank among all 12 new agents	Rank in monotherapy-only subset	W1 tier	Rank change after exclusion	Interpretation
Sevabertinib	0.860	1	1	High	0	Remained highest-ranked new agent
Zongertinib	0.840	2	2	High	0	Remained second-ranked new agent
Taletrectinib	0.736	4	3	Moderate	+1	Became third-ranked after exclusion of combination-based agents
Sunvozertinib	0.708	5	4	Moderate	+1	Upper-priority position preserved
Ensartinib	0.547	7	5	Moderate	+2	Mid-tier position preserved
Vimseltinib	0.504	9	6	Moderate	+3	Mid-tier position preserved
Tovorafenib	0.397	11	7	Low	+4	Low-tier position preserved
Mirdametinib	0.080	12	8	Low	+4	Remained lowest-ranked outlier

[a]Combination-based approvals excluded from this sensitivity analysis were Avutometinib (+ Defactinib), Defactinib (+ Avutometinib), Inavolisib (+ Palbociclib/Fulvestrant), and Lazertinib (+ Amivantamab). W1 scores were not recalculated; rankings were re-expressed after exclusion of explicitly combination-based approvals. In the full 12-agent cohort, the W1 tier distribution was 3 High / 6 Moderate / 3 Low; in the monotherapy-only subset, the distribution became 2 High / 4 Moderate / 2 Low. The principal prioritization extremes were preserved: Sevabertinib and Zongertinib remained the two highest-ranked newly approved agents, and Mirdametinib remained the lowest-ranked outlier.

Table S4.4. Exploratory FAERS-based post-marketing contextual summary aligned to the ToxPi ranking order

Drug (ToxPi order)	Brand	Approval date	Total reports	Hepatic cases	Cardiac/QT cases	Broader cardiac cases	wToxPi tier	Contextual interpretation
Imatinib (REF)	Gleevec/Glivec	2001-05-10	65840	1601	222	2435	High	Historical reference only; long exposure window makes direct comparison with newly approved agents inappropriate.
Sevabertinib	Hyrnuo	2025-11-19	5	0	0	0	High	Sparse early FAERS set; no retrieved hepatic or strict QT-specific PTs.
Zongertinib	Hernexeos	2025-08-08	71	7	0	3	High	Hepatic reporting present; no strict QT-specific PTs; broader cardiac PTs present.
Lazertinib	Lazcluze	2024-08-19	555	20	1	18	High	Combination-confounded by amivantamab; hepatic reporting present; 1 strict QT-specific PT.
Taletrectinib	Ibtrozi	2025-06-11	64	18	0	5	Moderate	Hepatic reporting present; no strict QT-specific PTs.
Sunvozertinib	Zegfrovy	2025-07-02	26	1	0	0	Moderate	Low-volume early FAERS; limited hepatic reporting; no strict QT-specific PTs.
Defactinib	Avmapki Fakzynja Co-Pack	2025-05-08	528	30	0	3	Moderate	Shared co-pack FAERS context with avutometinib; attribution cannot be separated.
Ensartinib	Ensacove	2024-12-18	35	5	1	4	Moderate	Hepatic reporting present; 1 strict QT-specific PT.
Avutometinib	Avmapki Fakzynja Co-Pack	2025-05-08	528	30	0	3	Moderate	Shared co-pack FAERS context with defactinib; attribution cannot be separated.
Vimseltinib	Romvimza	2025-02-14	427	63	0	4	Moderate	Early post-marketing reporting shows a strong hepatic signal; no strict QT-specific PTs.
Inavolisib	Itovebi	2024-10-10	331	12	1	3	Low	Combination-confounded by palbociclib/fulvestrant; hepatic reporting present; 1 strict QT-specific PT.
Tovorafenib	Ojemda	2024-04-23	258	11	0	4	Low	Hepatic reporting supports the endpoint-level hepatic signal despite low composite rank.
Mirdametinib	Gomekli	2025-02-11	76	2	0	3	Low	Low-level hepatic reporting only; no strict QT-specific PTs.

[a]PT: MedDRA Preferred Term. Observation windows ran from each compound's FDA approval date to 31 March 2026; for imatinib, the contextual reference window ran from 10 May 2001 to 31 March 2026. Hepatic cases were counted using DILI-compatible preferred terms in case-level line listings. Strict Cardiac/QT cases were limited to QT-specific preferred terms. Broader cardiac cases were recorded separately to avoid overstating direct hERG/QT concordance. Retrieved line listings may include both single-agent and combination-associated reports. Accordingly, Table S4.4 should be interpreted as indexed-compound FAERS context rather than monotherapy-attributable validation, with the greatest regimen-level confounding expected for Lazertinib, Inavolisib, Avutometinib, and Defactinib. Avutometinib and Defactinib share the same co-pack regimen-level FAERS context; attribution cannot be separated between the two components. Lazertinib and Inavolisib remain separate rows to preserve the drug-level structure of the ToxPi table, but their FAERS interpretation remains substantially combination-confounded. FAERS observations are exploratory and contextual only. They do not provide denominator-based risk estimates and should not be interpreted as formal validation or as evidence of absence of clinical risk when report counts are low.
